# A Single Zidovudine (AZT) Administration Delays Hepatic Cell Proliferation by Altering Oxidative State in the Regenerating Rat Liver

**DOI:** 10.1155/2017/8356175

**Published:** 2017-04-05

**Authors:** Armando Butanda-Ochoa, Diego Rolando Hernández-Espinosa, Marisela Olguín-Martínez, Lourdes Sánchez-Sevilla, Mario R. Rodríguez, Benito Chávez-Rentería, Alberto Aranda-Fraustro, Rolando Hernández-Muñoz

**Affiliations:** ^1^Departamento de Biología Celular y Desarrollo, Instituto de Fisiología Celular, Universidad Nacional Autónoma de México (UNAM), 04510 Ciudad de México, Mexico; ^2^Departamento de Neurodesarrollo y Fisiología, Instituto de Fisiología Celular, Universidad Nacional Autónoma de México (UNAM), 04510 Ciudad de México, Mexico; ^3^Departamento de Patología Instituto Nacional de Cardiología “Ignacio Chávez”, 14080 Ciudad de México, Mexico

## Abstract

The 3′-azido-3′-deoxythymidine or Zidovudine (AZT) was the first antiretroviral drug used in the treatment of HIV patients, which has good effectiveness but also hepatotoxic side effects that include cell cycle arrest and oxidative/nitrative mitochondrial damage. Whether such an oxidative damage may affect the proliferative-regenerative capacity of liver remains to be clearly specified at doses commonly used in the clinical practice. In this study, we described the oxidative-proliferative effect of AZT administered at a common clinical dose in rat liver submitted to 70% partial hepatectomy (PH). The results indicate that AZT significantly decreased DNA synthesis and the number of mitosis in liver subjected to PH in a synchronized way with the promotion of organelle-selective lipid peroxidation events (especially those observed in plasma membrane and cytosolic fractions) and with liver enzyme release to the bloodstream. Then at the dose used in clinical practice AZT decreased liver regeneration but stimulates oxidative events involved during the proliferation process in a way that each membrane system inside the cell preserves its integrity in order to maintain the cell proliferative process. Here, the induction of large amounts of free ammonia in the systemic circulation could become a factor capable of mediating the deleterious effects of AZT on PH-induced rat liver regeneration.

## 1. Introduction

According to the World Health Organization at the end of 2015 there were 36.7 million persons infected with HIV in the world (see Progress Report at http://www.who.int/hiv/data/en/) and 1.1 million people are dead because of HIV. The common treatment for those patients is the administration of antiretroviral nucleosides (18.2 million of patients were medicated with them in the middle of 2016 and that number is expected to increase to 30 million in 2020). Among them, the 3′-azido-3′-deoxythymidine or Zidovudine (AZT) was the first antiretroviral drug used [1, 2] and its therapeutic effect includes the blockade of the cytopathic effect and the inhibition of the viral reverse transcriptase activity [3–5]. This antiretroviral agent shows good effectiveness but also unfortunate (concentration and time of exposure dependent) side effects. The specific molecular structure of AZT can contribute to carcinogenesis causing DNA damage. The initial phosphorylation to give AZT 5′-monophosphate is performed by thymidine kinase 1 (TK1), constituting the key regulatory step in AZT metabolism and the activity of this cytosolic TK1 is dependent on the cell cycle progression (in comparison to the mitochondrial TK2 that is not relevant in cell proliferation) [6]. The AZT-5′-triphosphate formed can be incorporated into nuclear DNA instead of the thymidine nucleotide [7, 8]. This DNA damage is repaired through the Nucleotide Excision Repair (NER) pathways [9] and it also involves cell cycle arrest by increasing the expression of phosphorylated checkpoint kinase 1 and 2 [10].

Once AZT is transported into the mitochondria [11, 12], the AZT-5′-triphosphate formed inside [8, 13] impairs the bioenergetics and increments H_2_O_2_ production, when glutamate/malate were used as substrates. Indicating a severe effect at the level of respiratory complex-I [14], particularly at the sub-complex-I*β* [15]. The enhanced H_2_O_2_ content induces a large increase in reactive oxygen species (ROS) and peroxynitrite production that causes single strand DNA breaks, lipid peroxidation, protein oxidation/nitration, and mitochondrial DNA (mtDNA) oxidation [16, 17]. Experimental evidence indicates that these prooxidative effects of AZT (or AZT-5′-triphosphate) are more related to mtDNA depletion than premature chain termination of mtDNA synthesis caused by inhibition of mitochondrial DNA-polymerase-*γ* [17–19]. AZT also increases lactate production but it is unclear whether it is due to poly-ADP ribose polymerase activation caused by ROS production, by direct inhibition of NADH oxidation into the mitochondria or both [16, 18]. In cardiac cells the ROS production caused by AZT also have epigenetic effects by altering the expression of 95 genes and reducing DNA methylation probably by reducing S-adenosylmethionine abundance that, in turns, reduces available substrates for new DNA methylation [19].

Based upon the aforementioned, hepatotoxicity is one of the most common adverse side effects associated with AZT involved in the oxidative/nitrative mitochondrial impairment already described, which also promotes fat accumulation by increasing fat synthesis and suppressing its degradation pathway [20] causing hepatocellular damage manifested as macro- and microvacuolar steatosis [21]. Whether such oxidative damage may affect one of the major intrinsic properties of the liver cells which is to proliferate, in order to regenerate the whole hepatic tissue, remains to be more clearly and widely understood particularly at doses commonly used in the clinical practice. In this regard, it is known that, depending on its concentration and the cell type experimentally used, AZT promotes cell cycle arrest and alters gene expression for TK1 [8, 22]. On the other hand, there is in vivo experimental evidence suggesting that lipid peroxidation (LP), induced by ROS, plays a role during the liver proliferative process [23]. In fact, it has been proposed that controlled peroxidative modifications of membranes could be playing a role in the early steps of liver regeneration and that a decrease in the magnitude or in the time-course of this partial hepatectomy (PH) induced an increase in lipid peroxidation (LP), as it occurs after administration of *α*-tocopherol, that could promote an early termination of the intracellular preparative events required for the replicative phase during the surgical-induced liver proliferation [23, 24].

Based on the anterior, it is possible that, at a dose commonly used in clinical practice, AZT may alter the proliferative potential of the liver and this may involve some selective alterations in the oxidative events that occurred during PH-induced rat liver regeneration. In vivo data presented here contribute to having a wider and closer approximation to understand the hepatotoxicity induced by a single clinically-used dose of AZT, especially with regard to how this antiretroviral alters the intrinsic capacity of the liver to regenerate and how such alterations may be related to other hepatocellular oxidative events. Therefore, in the present study, we described the liver oxidative-proliferative effects of a single clinical AZT administration to rats subjected to 70% PH.

## 2. Methods

### 2.1. Materials

The [^3^H]-Thymidine (specific activity 2 Ci/mmol) was purchased from Perkin Elmer. Other reagents were obtained from Sigma Chemical Co. (St Louis MO) or JT Baker Chemicals.

### 2.2. Animals and Treatments

Male Wistar rats (of ~250 g weight) were fed ad libitum and maintained under a 12-hour light/dark period. Thereafter the animals were treated according to the next experimental groups: Sham, Sham + AZT, PH alone, and PH + AZT. The Sham (control) group were the laparotomized rats that received the AZT vehicle (0.9% NaCl) immediately after surgery and the Sham + AZT group were orally administered (using a gastric cannula) with 5 mg/Kg of body-weight AZT (a dose commonly used in the clinical practice). The PH group were those animals subjected to two-thirds (~70%) liver resection as described by Higgins and Anderson [25]. The PH + AZT group were those animals that were also orally administered with a single dose of 5 mg/Kg of body-weight AZT. Each experimental group comprised four animals that were independently analyzed per experimental time-point (*n* = 4). All surgical procedures were performed at about 9-10 AM, and rats were euthanized at 6, 12, 24, 48, or 72 h after surgery always under sedation with an overdose of sodium pentobarbital. All procedures were done in accordance with the* Mexican Federal Regulations for Animal Care and Experimentation *(Ministry of Agriculture, SAGARPA, NOM-062-ZOO-1999).

### 2.3. Isolation of Subcellular Fractions

Around 4 g of liver tissue was homogenized in 8 mL of Buffer A (225 moles/L sucrose, 10 mmol/L Tris-HCl, and 0.3 mmol/L EDTA, pH 7.4; thereafter, the homogenate was centrifuged at 1,800 g 15 min at 4°C. From here, the mitochondrial, cytosolic, and microsomal fractions were obtained and purified as described in detail by Aguilar-Delfín et al. [23]. To isolate the plasma membranes, this subcellular fraction was achieved by centrifuging through Percoll gradient, as described by Loten and Redshaw-Loten [26]. Finally, the nuclear fraction was isolated according to Sindić et al. [27]; briefly: 4 g of liver tissue was homogenized in 8 mL of buffer B (10 mmol/L HEPES pH 7.5, 5 mmol/L MgCl_2_, 25 mmol/L KCl); then, 1.4 mL of buffer C (10 mmol/L HEPES pH 7.5, 2 mmol/L MgCl_2_, and 2.4 mol/L sucrose) was added and mixed by inversion, followed by addition of another 26 mL of buffer D (10 mmol/L HEPES pH 7.5, 2 mmol/L MgCl_2_, and 2.3 mol/L sucrose). The whole mixture was placed on 7.5 mL of cold buffer D and spun at 120,000 g for 45 min. The final pellet was resuspended in 0.5 mL of buffer A (10 mmol/L HEPES pH 7.5, 2 mmol/L MgCl_2_ 0.25 mol/L sucrose). All isolated subcellular fractions were kept at −20°C until used.

### 2.4. Parameters Indicative of Liver Cell Proliferation

The cytosolic activity of thymidine kinase (TK) was assayed according to Sauer and Wilmanns [28]; briefly: cytosolic samples were incubated with a reaction mixture consisting in 0.2 mol/L Tris-HCl pH 8.0, 10 mmol/L EDTA, 20 mmol/L MgCl_2_, 40 mmol/L ATP, 450 *μ*mol/L thymidine, and 4.5 *μ*Ci [^3^H]-Thymidine for one hour at 37°C. After stopping the reaction, samples were spun and the supernatant was filtered (with Grade DE81 ion exchange DEAE-cellulose Whatman paper) and washed with 1 mM ammonium formate. The filter papers were dried and placed in 10 mL of Tritosol scintillation liquid and counted for dpm. Mitotic index was assessed with an optical microscope (Olympus, CH-30) taking into account the number of mitotic cells in 20 microscopic fields with a 40x objective.

### 2.5. Caspase-3 Activity Assay

After treatment, the tissue was lysed in Caspase Assay Buffer (50 mmol/L HEPES pH 7.4, 100 mmol/L NaCl, 0.1% CHAPS, 1 mmol/L EDTA, 10% glycerol, and 10 mmol/L DTT). Equal amounts of homogenate extract (300 *μ*g of protein) from each sample were analyzed. The assay was carried out by adding 100 *μ*mol/L of Ac-DEVD-AMC (Sigma Chemical Co.) at 37°C. The amount of fluorescent product was monitored continuously for 60 min with a spectrofluorometer (FLx800 BIO-TEK Instruments, Winooski, VT, USA) at an excitation wavelength of 355 nm and an emission wavelength of 460 nm [29]. Data were analyzed using the KC JUNIOR software (BIO-TEK Instruments, Winooski, VT, USA), normalized to fluorescence levels in vehicle-treated cells, and expressed as relative units of fluorescence (RUF).

### 2.6. Parameters Indicative of Oxidant Stress in Lipids and Proteins

The amount of some LP by-products, mainly malondialdehyde (MDA), conjugated dienes, and the protein carbonyl content (protein oxidation) were determined as previously reported, in detail [30]. In the case of the protein-attached carbonyl groups (oxidized proteins), the denaturalized proteins with trichloroacetic acid were stained with 2,4-dinitrophenylhydrazine and further precipitated with 6 mol/L guanidine dissolved in 20 mmol/L KH_2_PO_4_ and further measured at 375 nm and finally calculated by its absorption coefficient, according to Levine et al. [31].

### 2.7. Determination of Blood Levels for MDA and Free Ammonia and the Serum Enzyme Activities

Heparin-anti-coagulated blood was obtained from the experimental groups, and the serum was rapidly separated. Aliquots of serum and red blood cells (RBC) package were placed in ice-cold perchloric acid (8% w/v, final concentration). In neutralized perchloric extracts, the levels of MDA [30], free ammonia [32], and those of urea [33] were determined. In serum, the following enzyme activities were quantified: lactic dehydrogenase (LDH; EC 1.1.1.27), alanine aminotransferase (ALT; EC 2.6.1.2), and ornithine carbamoyltransferase (OCT; EC 2.1.3.3) by the methods described elsewhere [33]. As to Arginase (ARG; EC 3.5.3.1), this enzymatic activity was quantified according to Iyamu et al. [34].

### 2.8. Statistics

All results expressed as means ± error standard (SE) were analyzed using two-way analysis of variance, followed by pairwise comparisons (Tukey's-test). For individual comparisons, statistical analysis was performed using unpaired Student's *t*-test. In all cases, *p* < 0.01 was considered to be statistically significant.

## 3. Results

### 3.1. Effect of AZT Administration on the Liver Proliferative Profile

**Figure 1:**
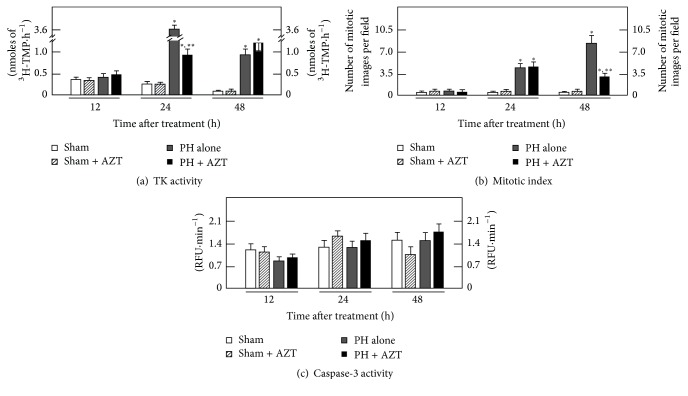
Effects of AZT administration on some parameters indicative of cell proliferation and apoptosis at various times after 70%-PH. Results are expressed as mean ± SE for four independent determinations per experimental point for panel (a). The activity of TK expressed as nmoles of formed [3H]-TMP·h^−1^·mg^−1^ of cytosolic protein, in panel (b). The number of mitotic cells per microscopic field, as well as the cytosolic activity of caspase-3 (apoptosis) expressed as Relative Fluorescence Units (RFU)·min^−1^·mg^−1^ of protein (panel (c)). Symbols for the experimental groups at the bottom of each figure. Statistical significance: ^*∗*^*p* < 0.01 against Sham-operated (control) rats, and ^*∗∗*^*p* < 0.01 versus the PH group.

In the proliferating tissue, the activity of cytosolic thymidine kinase 1 (TK1) usually increases to provide thymidine triphosphate for its incorporation during DNA synthesis. Here, the PH represented a physical and chemical stimulus for cell proliferation that leads TK1 activity to reach its maximum value at 24 h after surgery (Figure 1(a)). The expression and activity of cytosolic TK1 is cell cycle dependent and its increment meant that hepatocytes population was prepared for DNA synthesis-phase and, a day after (48 h after surgery), the whole cell cycle process was culminated producing a maximal number of mitotic cells (Figure 1(b)). After PH, the administered AZT (PH + AZT) induced a significant decrease (~3-fold) on the 24 h activity peak of TK1 that was sustained 24 h more and finally reached the basal levels of the Sham and Sham + AZT control groups at 72 h after surgery (data not shown); therefore, AZT significantly decreased the activity of TK1, hence promoting a partial inhibition on DNA synthesis. The same happened with the number of mitotic cells as well (Figure 1(b)); the rate of mitotic images was very scanty in the sham-control group, which was unmodified by the treatment with AZT. On the contrary, in animals subjected to PH and administered with AZT (PH + AZT), we noted an almost 3-fold decrease in cell mitosis (48 h after surgery) when compared with the group of PH alone (Figure 1(b)). This partial inhibition of rat liver regeneration induced by the administration of AZT to PH-rats was not apparently accompanied by a stimulated apoptosis, since the cytosolic caspase-3 activity remained without significant changes in those PH-animals treated with AZT (Figure 1(c)).

### 3.2. Effect of AZT Administration on the Liver Profile for Lipoperoxidative Events and Oxidation of Proteins in Subcellular Fractions

**Figure 2:**
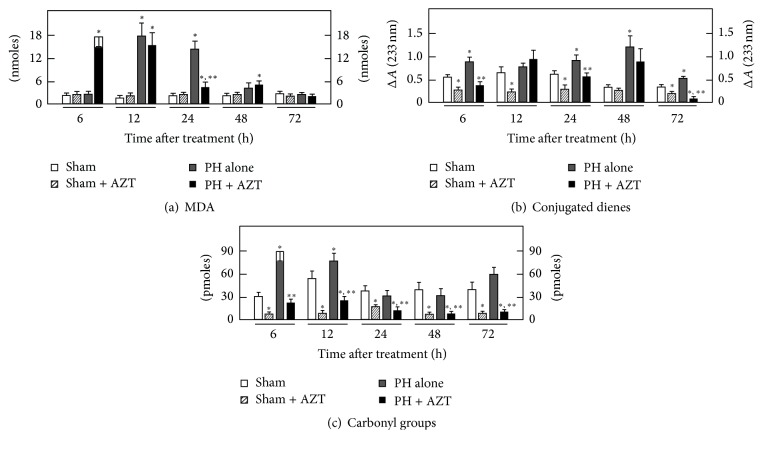
Effects of AZT on parameters indicative of oxidant stress in plasma membranes obtained from livers at various times after 70% PH. Results are expressed as mean ± SE for four independent determinations per experimental point as nmoles·mg^−1^ of protein in panels (a) and (b), or in pmoles·mg^−1^ of protein (panel (c)). Symbols for the experimental groups at the bottom of each figure and the statistical significance as indicated in Figure 1.

The AZT promotes oxidative stress that alters cell physiology in different ways. Experimental evidence has associated lipid peroxidation to the proliferative process during liver regeneration [23]; the results obtained here indicate that oral administration of AZT alters the pattern of lipid peroxidation according to each specific cellular fraction isolated from livers submitted to a proliferative stimulus such as 70% PH. In plasma membranes, LP (MDA and conjugated dienes) significantly initiated at 12 h after surgery and then decreased over time, while AZT administration induced an earlier LP (at 6 h after surgery) that was maintained until 12 h after surgery and then decreased thereafter (Figure 2(a)). The magnitude of MDA production is expected to correlate with conjugated dienes content, since the double conjugated bonds located along the carbon chains of lipids represent reactive (oxidable) sites for oxygen peroxides [35, 36]; therefore, when the conjugated dienes are consumed by reacting with ROS-derived peroxides, the amount of MDA and other final LP by-products will consequently increase. During the times considered in this study, control plasma membranes exhibited a basal conjugated dienes content which was significantly increased by PH along the times tested (Figure 2(b)). On the contrary, those PH-rats treated with AZT depicted lower values of conjugated dienes in plasma membranes, when compared with the group of PH alone (Figure 2(b)). Here, it should be noticed that, at 6 or 12 h after surgery, when the rate of MDA was high for plasma membranes in the PH + AZT group, the corresponding conjugated dienes levels were decreased in this experimental group.

The content of carbonyl groups indicates the rate of protein oxidation associated with an oxidative surround. In plasma membranes, administration of AZT after PH significantly decreased the levels of carbonyl groups through the times studied compared to those in the PH alone group, where we found an increase at 6 h, which diminished up to 48 h, returning to control values thereafter (Figure 2(c)). Together, these data indicated that in plasma membrane AZT influences the occurrence of oxidative events, mainly in the rate of LP, which occurred at earlier times after surgery rather than changing the magnitude of protein oxidation as a response to liver cell proliferation.

**Figure 3:**
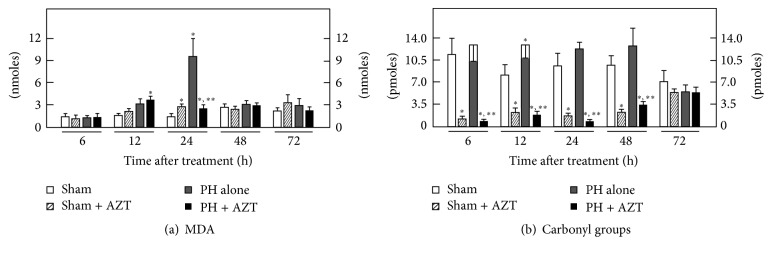
Effects of AZT on parameters indicative of oxidant stress in the cytosolic fraction obtained from livers at various times after 70% PH. Results are expressed as mean ± SE for four independent determinations per experimental point as nmoles·mg^−1^ of protein in panels (a) and (b), or in pmoles·mg^−1^ of protein (panel (c)). Symbols for the experimental groups at the bottom of each figure and the statistical significance as indicated in Figure 1.

Cytosolic LP was maximally increased showing a major peak at 24 h after surgery that returned to control values thereafter. AZT administration practically avoided the PH-induced increase in cytosolic LP (Figure 3(a)). When the amount of carbonyl groups was examined in the liver cytosolic fractions from PH-rats, we detected only a modest transient increase of oxidized proteins at 12 h after PH. On the contrary, AZT administration elicited a drastic protective effect on cytosolic protein oxidation in Sham-operated control animals and, when administered to PH-rats, the nucleoside was also able to significantly decrease the content of protein's carbonyl groups in the cytosolic fraction obtained from these animals (Figure 3(b)).

**Figure 4:**
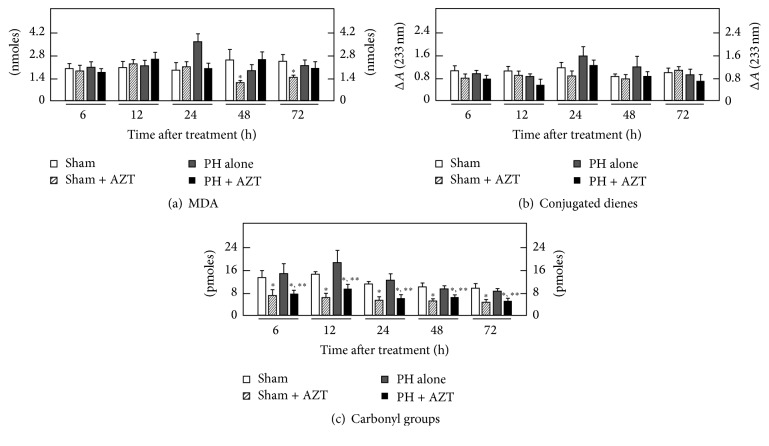
Effects of AZT on parameters indicative of oxidant stress in isolated mitochondria obtained from livers at various times after 70% PH. Results are expressed as mean ± SE for four independent determinations per experimental point as nmoles·mg^−1^ of protein in panels (a) and (b), or in pmoles·mg^−1^ of protein (panel (c)). Symbols for the experimental groups at the bottom of each figure and the statistical significance as indicated in Figure 1.

As to mitochondrial LP (MDA and conjugated dienes), PH had not significant changes in this parameter and neither had AZT administration after PH at 24 h (Figure 4(a)), and we did not find significant changes in the content of conjugated dienes in these experimental groups (Figure 4(b)). However, the amount of mitochondrial protein carbonyl groups was indeed modified by AZT administration (Figure 4(c)); whereas PH alone did not induce significant changes in this parameter, AZT administration promoted significantly lower levels of mitochondrial carbonyl groups along all the times tested (Figure 4(c)). Data indicated that under proliferative conditions AZT did not promote evident oxidative events (not for lipids or for proteins) in the hepatic mitochondrion.

**Figure 5:**
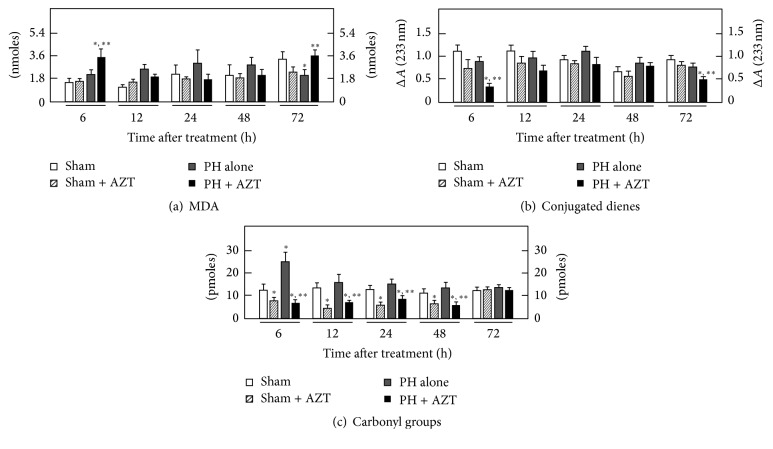
Effects of AZT on parameters indicative of oxidant stress in microsomes (Endoplasmic Reticulum) obtained from livers at various times after 70% PH. Results are expressed as mean ± SE for four independent determinations per experimental point as nmoles·mg^−1^ of protein in panels (a) and (b), or in pmoles·mg^−1^ of protein (panel (c)). Symbols for the experimental groups at the bottom of each figure and the statistical significance as indicated in Figure 1.

In the microsomal fraction, PH alone did not increase the rate of LP, as assessed by MDA content, but AZT enhanced this LP by-product at 6 and 72 h after surgery in animals subjected to PH, when compared to those with PH alone (Figure 5(a)). Whereas PH alone did not significantly modify microsomal content of conjugated dienes, AZT changed this parameter at the same experimental times (6 and 72 h after surgery, resp.) but in an opposite manner (Figure 5(b)). The carboxyl content in the microsomal fraction obtained from PH-animals only showed an early significant increase (6 h after surgery). Despite the scanty early (6 h) effects of PH in this parameter, the concomitant administration of AZT strongly decreased the rate of oxidized proteins in both sham-control and PH along the times tested (Figure 5(c)).

**Figure 6:**
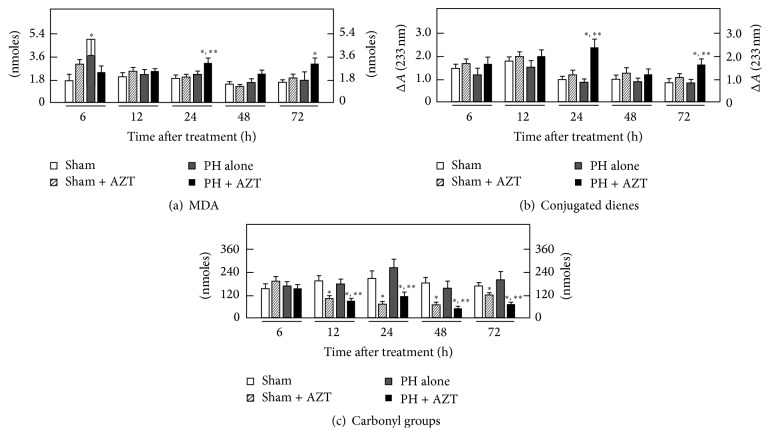
Effects of AZT on parameters indicative of oxidant stress in nuclei obtained from livers at various times after 70% PH. Results are expressed as mean ± SE for four independent determinations per experimental point as nmoles·mg^−1^ of protein in panels (a) to (c). Symbols for the experimental groups at the bottom of each figure and the statistical significance as indicated in Figure 1.

Regarding the nuclear fraction, PH alone promoted an early increase (6 h) in the amount of MDA, which rapidly decreased thereafter (Figure 6(a)) and was not accompanied by significant changes in the nuclear content for conjugated dienes (Figure 6(b)). The AZT treatment after PH elicited a significant lipid peroxidative effect at 24 and 72 h, respectively (Figure 6(a)), which was also associated with an active formation of conjugated dienes at the same experimental post-PH times (Figure 6(a)).

In agreement with the absence of effects on parameters indicative of LP, PH alone did not induce significant changes in the nuclear content of protein carbonyl groups (Figure 6(c)). On the contrary, in this subcellular fraction, the administration of AZT also exerted an important “protective” effect on nuclear protein oxidation in either control (Sham + AZT) or hepatectomized (PH + AZT) animals, practically at all postsurgery times tested (Figure 6(c)). It is noteworthy to mention that these organelle-selective peroxidation changes, already described, were not associated with apoptotic events as indicated by caspase-3 activity, which was not changed by either PH alone or AZT administration (Figure 1(c)).

### 3.3. Effect of AZT Administration on the Serum Enzyme Activity

**Figure 7:**
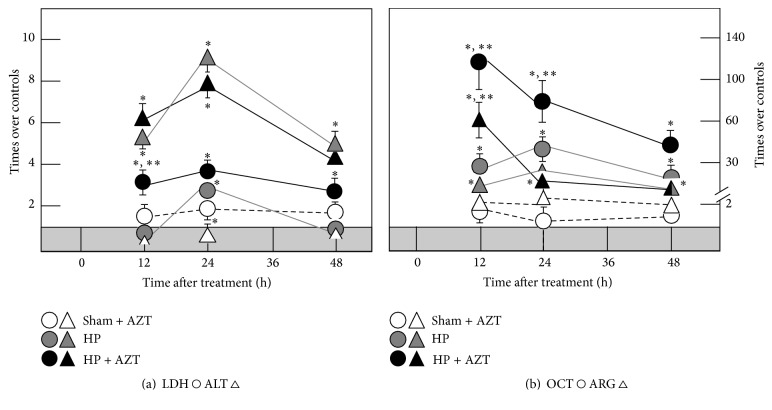
Effects of AZTon serum enzyme activities in samples obtained at several times after 70% PH. Results of serum enzyme activities are expressed as mean ± SE mean of four independent determinations per experimental point. In both panels, the shadowed horizontal bar represents the range of serum enzyme activities for controls (Sham): ALT 20 ± 3, LDH 570 ± 90, OCT 2.1 ± 0.3, and Arginase, 4.5 ± 0.7 IU/L. Symbols for the experimental groups at the bottom of each figure. Statistical significance as indicated in Figure 1.

Clinical practice uses some enzyme serum activity levels as indicative of liver damage, despite their levels inside the organ are not changing [37]. As previously reported [38] after PH, the serum activities of LDH, ALT, and OCT were significantly increased in a synchronized manner to DNA synthesis and mitosis showing the main peaks at 24 h after surgery and completely normalizing three days after the surgery (Figures 7(a) and 7(b)). When AZT was administered, the serum LDH activity was indeed early increased (12 h) and remained high in animals subjected to PH, whereas the profile for serum ALT induced by PH was not significantly modified by the nucleoside administration (Figure 7(a)). Regarding the OCT activity, the AZT induced an early (12 h) and drastic increase of the serum activity of this enzyme, which decreased thereafter, in a different pattern when compared to PH-animals receiving only the vehicle (Figure 7(b)). Moreover, another hepatic enzyme also involved in the cytoplasmic component of the urea cycle, namely, ARG, also shown changes in its “release” after PH. The partial removal of the liver was also accompanied by gradual increase in the serum activity of ARG, peaking at 24 h after PH, which was also altered by the administration of AZT to these animals in a similar fashion than that recorded for the activity of OCT (Figure 7(b)). Therefore, the administration of the nucleoside readily affected the magnitude and time-course of the pattern of serum enzyme activities induced by PH, which was not recorded in the control animals. As a whole, AZT indeed modified the coordinated pattern of enzyme's release elicited by the PH.

### 3.4. Effect of AZT Administration on Blood Levels of MDA, Ammonia, and Urea

**Figure 8:**
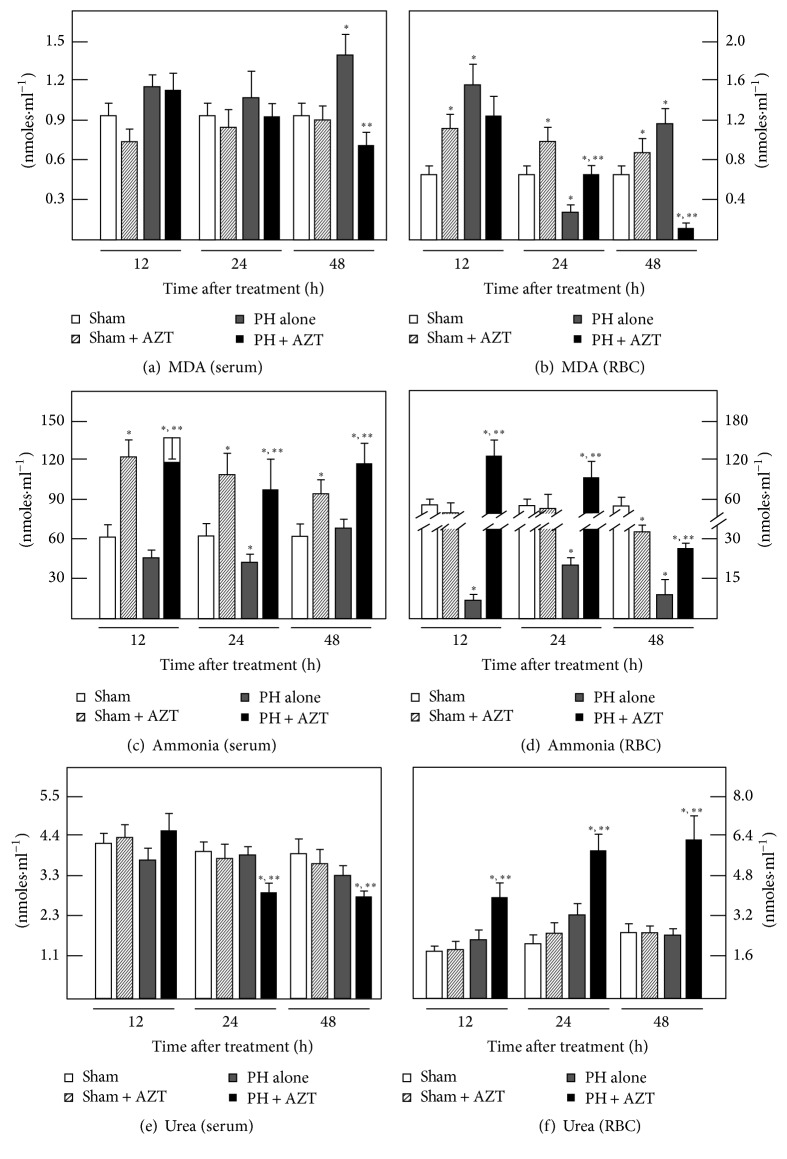
Effects of AZTon blood levels for MDA, ammonia, and urea in samples obtained at several times after 70% PH. Results of serum enzyme activities are expressed as mean ± SE mean of four independent determinations per experimental point. Symbols for the experimental groups at the bottom of each figure. Statistical significance as indicated in Figure 1.

As another parameter of liver damage, we looked for possible peroxidative events in blood compartments, serum, and RBC. In serum, PH induced a discrete increase in the MDA level (compared to control groups) only at 48 h, and the administration of AZT showed a lower value, but similar to the control ones at that time. In RBC, PH also elicited a first augmentation of MDA at 12 h, which decayed in the next twelve hours after PH, raising again at 48 h after surgery (Figures 8(a) and 8(b)). The AZT administration promoted a distinct MDA pattern in RBC from PH-animals, represented by a progressive decrement of this LP by-product throughout the times tested, even reaching MDA levels far lower than that of controls (Figures 8(a) and 8(b)). It is interesting to notice that the presence of AZT in RBC may induce oxidative events temporarily independent from liver proliferation, since the Sham + AZT group showed a sustained significant enhancement of oxidative by-products levels when compared with Sham controls alone throughout this range of posttreatment time.

Blood ammonia levels can reflect liver dysfunction, which results from a compromised hepatic handling for this cation, that is, liver injury (Figures 8(c) and 8(d)). The serum levels of ammonia were maintained within the normal range and even decreased at 24 h after PH (Figure 8(c)). During PH-induced rat liver regeneration, RBC levels of ammonia almost disappeared, suggesting that an increment in blood ammonia may be an adverse effect on the “primed” liver cell proliferation promoted by PH (Figure 8(d)). In fact, a single administration of AZT induced a drastic increase of ammonia levels in both serum and RBC from sham-control and PH-rats (Figure 8(d)). Therefore, the partial inhibition in PH-induced rat liver regeneration caused by treatment with AZT was also associated with a clear hyperammonemia present in these animals, besides the alterations found in the oxidant status (Figures 2to 6). In order to determine whether the AZT-induced increase in blood levels of ammonia was due to a defective liver ureagenesis, we also measured this nitrogen by-product in the blood compartments from our experimental groups (Figures 8(e) and 8(f)). Despite the fact that AZT administration elicited high blood levels, the nucleoside did not significantly modify serum and RBC levels of urea in control-laparotomized rats (Figures 8(e) and 8(f)). On the other hand, whereas PH alone did not change levels of blood urea, the administration of AZT significantly diminished serum levels of urea in PH-animals at 24 and 48 h after surgery (Figure 8(e)), contrasting with an unexpected and drastic accumulation of urea in RBC from the PH + AZT group (Figure 8(f)). Indeed, these results indicated that animals subjected to PH and administered with AZT had similar values for urea per mL of whole blood (3.7 ± 0.5 against 3.1 ± 0.4 *μ*moles·mL^−1^ of blood, at 24 h after PH). Therefore, the augmented levels of ammonia in the blood of animals treated with a single AZT dose did not seem to be due to an ineffective liver production of urea induced by the nucleoside.

## 4. Discussion

Liver regeneration must undergo changes in major metabolic pathways in order to achieve DNA replication, cell division, and restitution of liver mass [39]. Hence, synthesis of thymidine triphosphate from thymidine by the cytosolic TK1 activity evidences an active DNA synthesis after PH; then the surgery represented a proliferative physical stimulus that promoted the DNA synthesis until a 24 h maximum peak which was followed by another mitosis peak a day later (Figures 1(a) and 1(b)). Here, AZT administration promoted a similar ~3-fold decrease on TK1 activity (associated with DNA synthesis) and on the number of mitosis (cell divisions). This effect on proliferative response is in accordance with a previous report [9] where it has been shown that, depending on its concentration, AZT arrests the hepatic cell cycle promoting phosphorylation of checkpoints 1 and 2 proteins that are involved in cell cycle regulation at the S-phase. Therefore, the mitotic rate occurred as an evident consequence of the surgical stimulus but at the dose administered here the azido-deoxynucleoside made the number of mitotic cell divisions significantly decreased (Figure 1(b)), due to a decreased DNA synthesis. However, it has to keep in mind that, under our experimental conditions, the AZT administration still allowed an evident TK1 activity compared to the control groups, which entailed a significant but discrete liver cell mitosis 24 h after the S-phase. Thus, at the clinical dose used, AZT deeply reduced the enzyme activity but did not induce its complete inhibition nor in the DNA synthesis or the mitotic cell division. Hence, under our experimental conditions, the cell cycle arrest induced by AZT may be partial, and further information must be generated in that direction. It is also interesting that the AZT-induced reduction on liver cell proliferation was not accompanied by apoptotic events mediated by caspases.

After PH, lipid peroxidative events are associated with and synchronized to proliferation but in an organelle-selective way [23]. The administration of AZT promotes an early (6–12 h) peroxidative stimulus that may represent a change in fluidity of plasma membrane [40] that prepares a posterior enzyme release to the extracellular space. In plasma membrane peroxidation of lipids is chemically preferred over proteins due to their structural double conjugated bonds that constitute reactive sites for peroxide species [36, 37]. Then, lipids of plasma membrane may represent constitutive scavengers that selectively avoid protein peroxidation. In the cytoplasmic space, where lipids and proteins are not organized and compacted as in intracellular membranes, their conjugated dienes do not have the same chemical potential to react with peroxide species. In the case of PH-induced rat liver regeneration, the LP events occurred only as a major transient event during the first 24 h of the proliferative process, and the rate of proteins oxidation was not significantly modified or even avoided when AZT was administered. Under these conditions, the effect of the administered AZT contrasted with the scheme observed for plasma membrane where AZT administration induced an early LP.

Apparently, the transient peroxidation occurred in cytosol and the change in plasma membrane fluidity (because of early LP) allows the release of active enzymes (Figure 7) that may constitute extrahepatic signals that may stimulate retrograde signals for some other organs that may contribute to the whole liver regeneration process as suggested by previous data obtained by our group [37, 38], where enzyme release under proliferative conditions was not associated with liver damage or necrosis. AZT seems to promote an early 6 h postsurgery change in plasma membrane fluidity, which was previous to the premature release of some liver enzymes (Figure 7) that may represent a signal involved in a decreased DNA synthesis and a lower mitotic response.

In the mitochondria, the proliferative conditions did not stimulate the peroxidative events (not for lipids neither for proteins) at a significant level even in the presence of AZT. Apparently, cell proliferation represents a stimulus that permits cell homeostasis to establish a control that prevails over the free radical-peroxidative uncontrolled chain reactions in order to preserve mitochondria structure and function, since it constitutes the main energy source for cell division. Therefore, it was not expected that AZT promoted the formation of any oxidative products in the mtDNA, such as the well-known 8-oxo-deoxyguanosine, previously reported [17].

The release into the blood of enzymes located at the mitochondrial compartment, such as OCT, did occur at high levels and at early times (before DNA synthesis and mitosis peaks) probably because of the high demand for extrahepatic retrograde signals. Besides, since the hepatic cells are being physically stimulated to proliferate and, so far, no ammonia production has been reported in blood cells, it is also possible that such mitochondrial enzyme release may be involved in some alterations of the urea cycle that generated the high levels of ammonia detected in the blood of PH and more notoriously in PH + AZT rats. Somehow the accumulated ammonia may be expulsed from the mitochondria to the bloodstream representing a possible source of the great ammonia levels observed in the serum and RBC of rats subjected to PH and particularly subjected to PH and AZT administration. However, a previous report from our group showed that despite the effect of PH in dragging out enzymes by in vitro perfusing the proliferating liver, the rate of urea cycle to remove ammonia is not adversely affected [32]. Moreover, despite AZT significantly increased blood levels of ammonia in control and PH-rats, this nucleoside did not reduce the amount of urea in the whole blood, but rather did change its distribution in the blood compartments (Figure 8). We ignore the physiological meaning of the differential distribution of these nitrogen-compounds in the blood compartments but, in the present study, we cannot explain the AZT-induced hyperammonemia as a consequence of a diminished liver capacity for removing this cation through forming urea. Another possibility is that AZT is altering glutamine metabolism by increasing its deamination to glutamic acid, with the corresponding production of ammonia overwhelming urea cycle; however, this hypothesis remains to be tested.

In the microsomal fraction, the activity of different metabolic enzymes depends on the integrity of membrane [41]. The increment induced by PH in lipid peroxidation correlates with a decrease in the activity of cytochrome P450E1 (CYP2E1) previously reported [42] and agrees with the gradual reduction of protein oxidation over the time. The clinical dose administration of AZT gradually reduced lipid peroxidation events and maintained far low the rate of protein oxidation during the proliferative process. In fact, the integrity of microsomal membrane and metabolic enzymes has to be preserved in order to generate the adequate reductive biochemical surround required to transform the azido-deoxynucleoside into the 3′-azido-3′-deoxy-5′-O-*β*-D-glucopyranuronosylthymidine (GAZT) and then to the final reduced 3′-amino-3′-deoxythymidine (AMT) by NADPH-cytochrome P450 reductase and cytochrome b5 [43–45].

Under proliferative conditions the integrity of the nucleus was practically preserved. Protein oxidation was very low in the presence of AZT. Then, it seems that AZT might exert a kind of “antioxidant” effect, mainly in this subcellular fraction. Indeed, in the presence of AZT, final LP by-products (MDA, for instance) were not completed and some conjugated double bonds were left unreacted as observed at 24 h when lipid peroxidation raised very discretely and conjugated dienes were above the control group receiving AZT. This would suggest that adequate conditions for cell proliferation demand the preservation of nuclear integrity.

Persistent slight or modest increments in blood ammonia level resulting from continuous intravenous infusion of the cation depressed hepatic TK1 and ornithine decarboxylase activities, as regenerative enzymes after 30% or 70% PH. Specifically, TK1 activity was similarly depressed by the large transient amounts of ammonia but it was less affected by the persistent smaller amounts [46]. These data strongly indicated that ammonia can exert deleterious effects on rat liver regeneration. In this study, administration of AZT to both control Sham-operated rats and PH-animals induced a drastic elevation of blood ammonia levels in either controls and PH-animals (Figures 8(c) and 8(d)). In this regard, despite the fact that AZT does not generate ammonia during its catabolism, the fact that this cation increases mainly in those animals undergoing PH can be considered as a noxious factor that is promoting adverse actions on the rat liver regeneration induced by PH.

## 5. Conclusion

The present model was designed to test the possible adverse effects of AZT administered at clinical dosing, as an attempt to resemble what happens with patients that need to be treated with antiretroviral drugs and also have a liver compromised to regenerate, as this can be expected in patients infected with HIV and coinfected with viral hepatitis (HBV or HCV). In this model, administration of a single AZT dose used here decreased parameters indicative of liver cell proliferation in animals subjected to 70% PH, which coincided with alterations in the PH-induced patterns of LP which are shown to be characteristics during rat liver regeneration induced by the surgery. Then the administered dose of AZT used in clinical practice decreased liver regeneration that correlated with a constant “antioxidant” effect on the rate of protein oxidation in most of the subcellular fractions tested, which highlights the importance of preserving the functional integrity of all those proteins involved in this cell proliferative process. Moreover, the induction of large amounts of free ammonia in the systemic bloodstream could become a factor capable of mediating the deleterious effects of AZT on PH-induced rat liver regeneration. The fact that the production of the ammonia-removing molecule, urea, was not significantly affected by the administration of AZT raises the need of exploring the effects of AZT on liver nitrogen metabolism that could be involved in the pharmacological effects of this nucleoside used in the antiretroviral therapy for patients undergoing HIV infection.
